# Supplementary material for: Multivariate prediction of mixed, multilevel, sequential outcomes arising from in vitro fertilisation
Source: Diagn Progn Res. 2021 Jan 21;5:2. doi: 10.1186/s41512-020-00091-2 (PMC7818923; doi:10.1186/s41512-020-00091-2)
Supplement: Supplementary file 1 — Additional file 1: Table A1. Estimated correlation matrix from the pretreatment joint model. Table A2. Estimated correlation matrix from the dynamic joint model. [file 41512_2020_91_MOESM1_ESM.docx]

|  | Number of oocytes | Fertilization rate | Embryo evenness | Embryo fragmentation | DET | LBE |
| --- | --- | --- | --- | --- | --- | --- |
| Number of oocytes | 1 | -0.62  (-0.67 to -0.56) | -0.01  (-0.08 to 0.06) | 0.03  (-0.03 to 0.09) | -0.09  (-0.16 to -0.02) | 0.16  (0.09 to 0.23) |
| Fertilization rate |  | 1 | -0.21  (-0.30 to -0.12) | -0.28  (-0.36 to -0.20) | 0.01  (-0.09 to 0.10) | 0.11  (0.01 to 0.21) |
| Embryo evenness |  |  | 1 | 0.87  (0.84 to 0.90) | -0.26  (-0.32 to -0.20) | 0.06  (-0.01 to 0.12) |
| Embryo fragmentation |  |  |  | 1 | -0.23  (-0.29 to -0.18) | 0.02  (-0.04 to 0.08) |
| DET |  |  |  |  | 1 | 0.04  (-0.02 to 0.10) |
| LBE |  |  |  |  |  | 1 |

Table A1: Estimated correlation matrix from the pretreatment joint model

|  | Number of oocytes | Fertilization rate | Embryo evenness | Embryo fragmentation | DET | LBE |
| --- | --- | --- | --- | --- | --- | --- |
| Number of oocytes | 1 | -0.58  (-0.64 to -0.52) | -0.01  (-0.31 to 0.36) | 0.31  (0.03 to 0.55) | 0.40  (0.13 to 0.63) | 0.36  (0.07 to 0.59) |
| Fertilization rate |  | 1 | 0.27  (-0.22 to 0.59) | 0.00  (-0.44 to 0.30) | 0.21  (-0.10 to 0.48) | 0.19  (-0.09 to 0.47) |
| Embryo evenness |  |  | 1 | 0.83 (  0.75 to 0.88) | -0.09  (-0.27 to 0.07) | 0.05  (-0.12 to 0.21) |
| Embryo fragmentation |  |  |  | 1 | -0.03  (-0.19 to 0.11) | 0.07  (-0.08 to 0.22) |
| DET |  |  |  |  | 1 | 0.34  (-0.03 to 0.62) |
| LBE |  |  |  |  |  | 1 |

Table A2: Estimated correlation matrix from the dynamic joint model
